# 

**Published:** 1887-09

**Authors:** 


### CONTENTS.

COMMUNICATIONS.

Temporary Teeth .................... 421
Treatment of Alveolar Abscess by the Jennings Method.................... 430
Specialty of Dentistry.................... 433
Address .................... 437

SELECTIONS.

Food Adulteration.................... 448
Medical Practitioners and Dentists .................... 453
Removal of Necrosed Bone by Irrigations with Weak Hydrochloric Acid.................... 453
Blood Poisoning .................... 457
The Prevention of Cholera Infantum and Kindred Diseases.................... 461
Bacteriology .................... 463
Cocoaine Anæsthesia.................... 465
Fatal Hæmorrhage From Tooth Extraction.................... 467
The Modern Tendency of Disease.................... 468
Hæmorrhage Diathesis.................... 469
SALMAGUNDI.................... 470

EDITORIAL.

Ohio State Dental Society.................... 471
New England Dental Society.................... 471
Dr. George Watt .................... 472
Obituary .................... 472

